# Umbilical cord-derived mesenchymal stem cells on scaffolds facilitate collagen degradation via upregulation of MMP-9 in rat uterine scars

**DOI:** 10.1186/s13287-017-0535-0

**Published:** 2017-04-18

**Authors:** Lu Xu, Lijun Ding, Lei Wang, Yun Cao, Hui Zhu, Jingjie Lu, Xin’an Li, Tianran Song, Yali Hu, Jianwu Dai

**Affiliations:** 1 Department of Obstetrics and Gynecology, Drum Tower Clinical Medical College of Nanjing Medical University, 321 Zhongshan Road, Nanjing, 210008 China; 2 Department of Obstetrics and Gynecology, the Drum Tower Hospital Affiliated to Nanjing University Medical School, 321 Zhongshan Road, Nanjing, 210008 China; 3 Institute of Genetics and Developmental Biology, Chinese Academy of Sciences, 3 Nanyitiao, Zhongguancun, Beijing, 100190 China

**Keywords:** UC-MSCs, Scaffolds, Uterine scars, MMP-9, Fertility

## Abstract

**Background:**

Severe injuries of the uterus may trigger uterine scar formation, ultimately leading to infertility or obstetrical complications. To date, few methods have adequately solved the problem of collagen deposition in uterine scars. Umbilical cord-derived mesenchymal stem cells (UC-MSCs) have shown great promise in clinical applications. The objective of this study was to investigate the effect of a scaffold/UC-MSCs construct on collagen degradation and functional regeneration in rat uterine scars following full-thickness excision of uterine walls.

**Methods:**

In order to establish a rat model of uterine scars, the uterine wall of approximately 1.0 cm in length and 0.5 cm in width (one-third of the uterine circumference) was excised from each uterine horn. A total of 128 scarred uterine horns from 64 rats were randomly assigned to four groups, including a PBS group (*n* = 32 uterine horns), scaffold group (*n* = 32 uterine horns), UC-MSCs group (*n* = 32 uterine horns) and scaffold/UC-MSCs group (*n* = 32 uterine horns) to investigate the effect of different treatments on the structure and function of uterine scars. PBS, degradable collagen fibres, UC-MSCs or UC-MSCs mixed with gelatinous degradable collagen fibres were injected into four pre-marked points surrounding each uterine scar, respectively. At days 30 and 60 post-transplantation, a subset of rats (*n* = 8 uterine horns) from each group was euthanized and serial sections of uterine tissues containing the operative region were prepared. Haematoxylin-eosin staining, Masson’s trichrome staining, and immunohistochemical staining for MMP-2, MMP-9, α-SMA and vWF were performed. Finally, another subset of rats (*n* = 16 uterine horns) from each group was mated with male rats at day 60 post-transplantation and euthanized 18 days after the presence of vaginal plugs to check numbers, sizes and weights of fetuses, as well as sites of implantation.

**Results:**

The scaffold/UC-MSCs group exhibited obvious collagen degradation compared with the other three groups. At day 60 post-transplantation, the number of MMP-9-positive cells in the scaffold/UC-MSCs group (25.96 ± 3.63) was significantly higher than that in the PBS group (8.19 ± 1.61, *P* < 0.01), the scaffold group (7.25 ± 2.17, *P* < 0.01) and the UC-MSCs group (8.31 ± 2.77, *P* < 0.01). The pregnancy rate in the scaffold/UC-MSCs group (10/16) was also significantly higher than that in the PBS group (2/16, *P* < 0.017), the scaffold group (1/16, *P* < 0.017) and the UC-MSCs group (3/16, *P* < 0.017).

**Conclusions:**

The scaffold/UC-MSCs system facilitated collagen degradation in uterine scars via upregulation of MMP-9, which was secreted by transplanted UC-MSCs, and promoted regeneration of the endometrium, myometrium and blood vessels in uterine scars. Furthermore, the scaffold/UC-MSCs-treated uterine scars showed nearly complete restoration of receptive fertility.

**Electronic supplementary material:**

The online version of this article (doi:10.1186/s13287-017-0535-0) contains supplementary material, which is available to authorized users.

## Background

The uterus provides an internal environment essential for embryo implantation and pregnancy maintenance [1]. Among women of reproductive ages, a normal endometrium undergoes approximately 400 cycles of proliferation, differentiation, shedding and regeneration without scarring. The basalis of the endometrium is permanent and works mainly through the activity of endometrial-intrinsic stem cells or progenitor cells for generation of a new functionalis in each menstrual cycle to prepare for blastocyst implantation. The processes involved in the cyclic scar-free repair of the endometrium, which represents the only example of scar-free repair in adult human tissue, include inflammation, endometrial-intrinsic stem cell proliferation and differentiation, as well as tissue remodelling [2–5]. When severe uterine traumas caused by caesarean section, curettage, myomectomy or infection impair the basalis of the endometrium, a loss of resident stem cells and the stem cell niche will take place. This triggers the excessive activation of fibroblasts and the subsequent continuous secretion of collagen, ultimately leading to formation of uterine scars. In return, excessive collagen deposition can impede the proliferation, differentiation and migration of uterine native cells, so the impaired uterine wall, especially the endometrium, presents collagenous scars [6, 7]. As a result, uterus factor infertility, recurrent miscarriage, placenta accreta and intrauterine growth restriction may take place [8–11].

Several strategies have been adopted for the treatment of uterine scars, however, few methods have adequately addressed the problem of collagen deposition [12–17]. A previous study by our group demonstrated that a collagen targeting vascular endothelial growth factor (VEGF) delivery system constructed by fusing a collagen-binding domain (CBD) to the N-terminal of native VEGF prompted remodelling of uterine scars including regeneration of blood vessels, endometrium and myometrium in a rat model [18]. We deduced that CBD-VEGF-induced collagen degradation may be due to increased vascularity and therefore increased access to matrix metalloproteinases in the scarred areas. Unfortunately, however, the clinical application of CBD-VEGF is currently restricted.

Mesenchymal stem cells (MSCs) have emerged as an attractive and promising tool for regenerative medicine. Increasing evidence supports that MSCs have an important role in repairing damaged tissue, not so much by means of cellular differentiation but rather via the secretion of a broad range of paracrine factors such as growth factors, cytokines and chemokines into their surroundings. These paracrine factors, which are anti-scarring, supportive and angiogenic, constitute the most biologically active component in the process of MSC-induced reconstruction of damaged tissue [19–21]. We also demonstrated that bone marrow derived-mesenchymal stem cells (BM-MSCs) loaded on collagen scaffolds improved regeneration of fresh wounds of the uterine wall via the secretion of fibroblast growth factor 2 (FGF2), insulin-like growth factor 1(IGF1) and vascular endothelial growth factor (VEGF) [22]. As a primitive population of MSCs between fetal and adult MSCs, umbilical cord-derived mesenchymal stem cells (UC-MSCs) have shown their prominent advantages in abundant supply, painless collection and high proliferative potential [23–28]. Accumulating evidence has shown that UC-MSCs facilitate the repair of bones, skin and peripheral nerves [29–31] and improve the function of organs following injury including the brain, kidneys and liver [32–34]. However, little is known about the effect of UC-MSCs on collagen degradation in uterine scars.

Degradable collagen fibres have been widely utilized in tissue engineering due to their abundance, low antigenicity, excellent biocompatibility and biodegradability [35–38]. During this process, degradable collagen fibres function not only as a highly organized, dynamically remodelled and three-dimensional framework for the structural support of tissues, but also as a functional guidance for cell adhesion, migration and differentiation [39, 40]. In the present study, we investigated the effect of UC-MSCs mixed with gelatinous degradable collagen fibres on collagen degradation and functional regeneration in rat uterine scars following full-thickness excision of uterine walls.

## Methods

### Establishment and treatments of a rat model of uterine scars

**Fig. 1:**
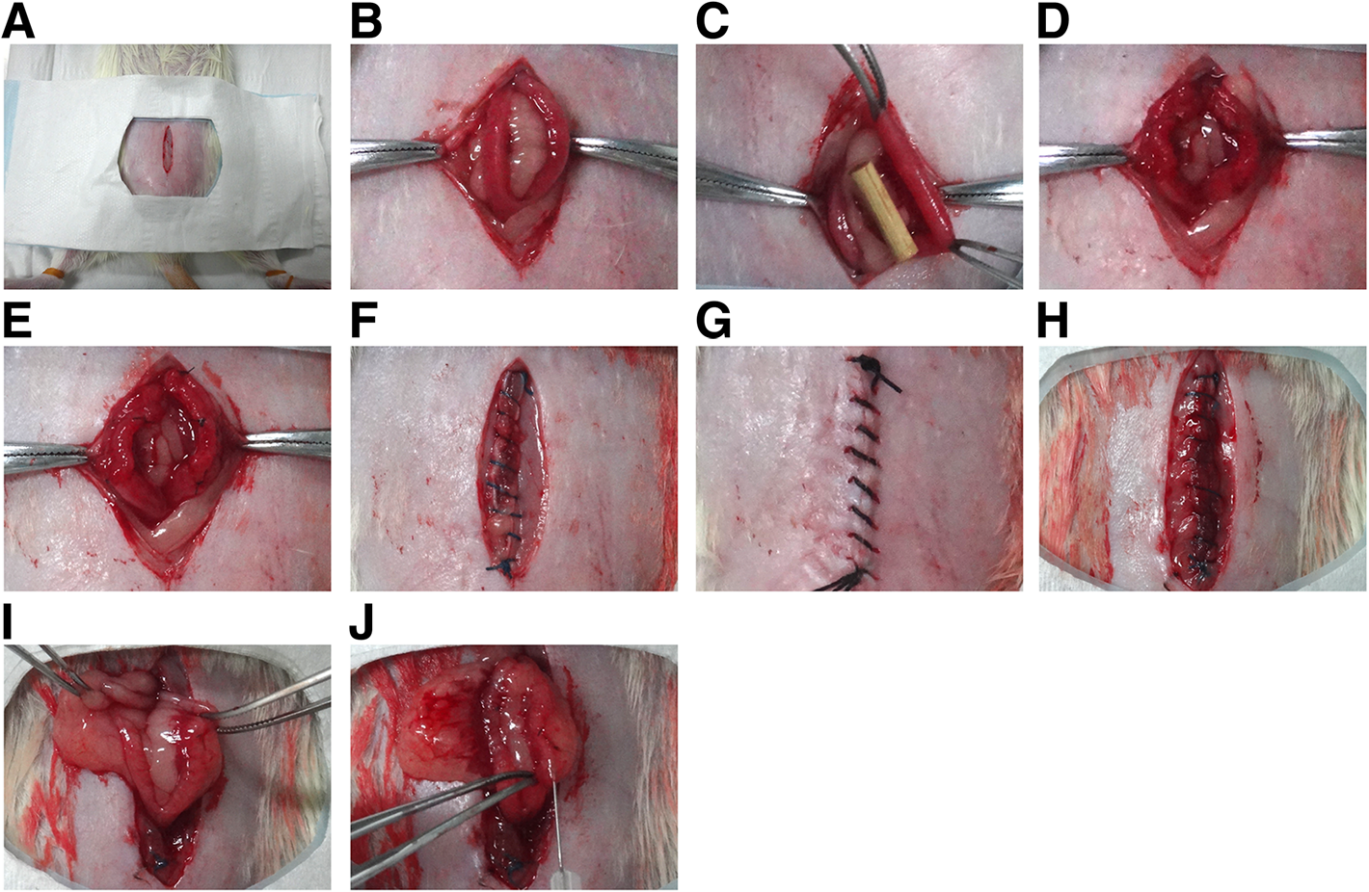
Establishment and treatments of a rat model of uterine scars. **a**-**b** After all rats were anesthetized by intraperitoneal injection of diazepam (50 mg/kg) and ketamine (50 mg/kg), a low abdominal midline incision was made to expose uterine horns. **c**-**d** The uterine wall of approximately 1.0 cm in length and 0.5 cm in width (one-third of the uterine circumference) was excised from each uterine horn, and the mesometrium was retained. **e** The four margins of the uterine wound were marked using a 6-0 nylon suture. **f**-**g** After rinsing the abdominal cavity with saline, the rectus fascia and skin were sutured with a 4-0 silk suture in an interrupted fashion. **h**–**i** Thirty days after full-thickness excision of uterine walls, a second incision was made in the abdominal wall of each rat to confirm scar formation. **j** PBS, degradable collagen fibres, UC-MSCs or UC-MSCs mixed with gelatinous degradable collagen fibres were injected into four pre-marked points surrounding each uterine scar

The animal experiments were conducted according to the guidelines of the Experimental Animals Management Committee (Jiangsu Province, China) and were approved by the Ethics Review Board for Animal Studies of the Drum Tower Hospital Affiliated to Nanjing Medical University. Female Sprague-Dawley (SD) rats weighing between 250 g and 300 g with consecutive 4-day estrous cycles were included in the experiments. A rat model of uterine scars following full-thickness excision of uterine walls was established as previously described [18]. Briefly, after all rats were anaesthetized by intraperitoneal injection of diazepam (50 mg/kg) and ketamine (50 mg/kg), a low abdominal midline incision was made to expose uterine horns (Fig. 1a, b). The uterine wall of approximately 1.0 cm in length and 0.5 cm in width (one-third of the uterine circumference) was excised from each uterine horn, and the mesometrium was retained (Fig. 1c, d). The four margins of the uterine wound were marked using a 6-0 nylon suture (Fig. 1e). After rinsing the abdominal cavity with saline, the rectus fascia and skin were sutured with a 4-0 silk suture in an interrupted fashion (Fig. 1f, g). All rats received intramuscular injection of penicillin twice a day for 3 days postoperatively.

As shown in Additional file 1: Figure S1, 128 scarred uterine horns from 64 rats were randomly assigned to four groups, including a phosphate-buffered saline (PBS) group (*n* = 32 uterine horns), scaffold group (*n* = 32 uterine horns), UC-MSCs group (*n* = 32 uterine horns) and scaffold/UC-MSCs group (*n* = 32 uterine horns) to investigate the effect of different treatments on the structure and function of uterine scars. Thirty days after full-thickness excision of uterine walls, a second incision was made in the abdominal wall of each rat (Fig. 1 h, i) and four different transplant components were injected into four previously marked points surrounding each uterine scar, respectively (Fig. 1j). For the PBS group, 50 μL PBS was injected per uterine horn. For the scaffold group, a mixture of 25 μL degradable collagen fibres and 25 μL PBS was injected per uterine horn. For the UC-MSC group, 10 × 10^5^ UC-MSCs in 50 μL PBS were transplanted per uterine horn. For the scaffold/UC-MSCs group, 10 × 10^5^ UC-MSCs in 25 μL PBS were mixed with 25 μL degradable collagen fibres, and the mixture were transplanted per uterine horn. The rectus fascia and skin were sutured and the rats were raised for the following experiments.

In addition, to track the transplanted UC-MSCs in uterine scars treated with UC-MSCs only (*n* = 4 uterine horns) or scaffold/UC-MSCs (*n* = 4 uterine horns), 10 × 10^5^ CM-Dil-labelled UC-MSCs in 50 μL PBS or 10 × 10^5^ CM-Dil-labelled UC-MSCs in 25 μL PBS and 25 μL degradable collagen fibres were injected into four previously marked points surrounding each uterine scar, respectively.

### Isolation and culture of UC-MSCs

Human umbilical cord tissues were obtained from full-term births after normal vaginal delivery at the Delivery Room of the Drum Tower Hospital Affiliated to Nanjing Medical University. Informed consent was obtained. The protocols for sampling human umbilical cord tissues were approved by the Ethics Review Board of the Drum Tower Hospital Affiliated to Nanjing Medical University. Briefly, fresh umbilical cord tissues (more than 10 cm in length) were collected in sterile boxes containing phosphate-buffered saline (PBS; Gibco, Grand Island, NY, USA) supplemented with 100 U/mL penicillin (Gibco), 100 μg/mL streptomycin (Gibco) on ice, rinsed several times with PBS supplemented with 100 U/mL penicillin (Gibco), 100 μg/mL streptomycin (Gibco) to remove blood clots and cut into 3 cm pieces. After removal of blood vessels, the residual tissue was chopped into 1 mm^3^ fragments with scissors. The fragments were incubated at 37 °C for 30 min in a humid atmosphere consisting of 5% CO_2_, and then complete growth medium consisting of LG-DMEM (Gibco) supplemented with 10% fetal bovine serum (FBS; Hyclone, Logan, UT, USA), 100 U/mL penicillin (Gibco), 100 μg/mL streptomycin (Gibco) was added. The umbilical cord tissues were removed after 14 days of culture. Cells were passaged when they reached 90% confluence. Human UC-MSCs at passages 3–5 were used for the following experiments.

### Flow cytometric analysis

Human UC-MSCs surface antigens were analysed by flow cytometric analysis at the fourth passage. In total, 5 × 10^5^ detached cells were incubated with 1% bovine serum albumin (BSA)/PBS (Gibco) for 30 min to block non-specific antigens. The cells were subsequently incubated in the dark with fluorescein isothiocyanate (FITC)-labelled anti-rat CD29 (AbD Serotec, Kidlington, UK), CD44 (AbD Serotec), CD90 (BD Pharmingen, San Diego, CA, USA), CD73 (BD Pharmingen), CD34 (Santa Cruz Biotechnology, Dallas, TX, USA), CD45 (Invitrogen, Carlsbad, CA, USA), HLA-DR (BD Pharmingen) and phycoerythrin (PE)-conjugated anti-rat CD105 (BD Pharmingen) at 4 °C for 30 min. The cells were washed twice with 1% BSA/PBS, resuspended in 200 μL 1% BSA/PBS and analysed by a flow cytometer (BD Biosciences, San Jose, CA, USA).

### Differentiation of UC-MSCs

The multi-lineage differentiation potential of human UC-MSCs was checked by adipogenic, osteogenic and neural-like differentiation assays at the fourth passage. Adipogenesis was induced by adipogenic induction medium (Gibco) for 14 days and confirmed by Oil red O staining to show intracellular lipid accumulation. Osteogenesis was induced by osteogenic induction medium (Gibco) for 28 days and calcium deposition was shown by Alizarin red staining. For neural-like differentiation, UC-MSCs seeded on poly-L-lysine-coated coverslips in a 24-well culture plate were treated with pre-induction medium containing 10^-7^ M all-trans-retinoic acid (ATRA; Sigma-Aldrich, St. Louis, MO, USA) and 10 ng/ml bFGF (Gibco) for 24 h and then with modified MNM medium for 36 h. The cells were co-incubated with the anti-NSE antibody (1:100, sc-292097, Santa Cruz Biotechnology) and the anti-NF-M antibody (1:100, sc-16143, Santa Cruz Biotechnology) to confirm their differentiation into neural-like cells.

### Preparation of scaffold/UC-MSCs

Degradable collagen fibres (collagen type I) were obtained from the Key Laboratory of Molecular Developmental Biology (Institute of Genetics and Developmental Biology, Chinese Academy of Sciences, Beijing, China) and were prepared as previously described [41]. In brief, one bottle of collagen fibres (20 mg) was dissolved in 3 mL PBS and pipetted repeatedly to form gelatinous scaffolds. The final concentration of degradable collagen fibres was 6.67 mg/mL. UC-MSCs were freshly mixed with degradable collagen fibres to form the scaffold/UC-MSCs construct prior to injection.

### Tracking of the transplanted UC-MSCs in uterine scars

Rats were sacrificed at day 30 post-transplantation. Uterine tissues containing the scarred areas were removed, embedded into optimal cutting temperature compound (OCT; Leica Microsystems, Nussloch, Germany) and frozen at −80 °C for 10 min. Sections of 5 μm were fixed in acetone and methyl alcohol (1:1) for 10 min, blocked with 3% BSA (Gibco) for 30 min and stained with the anti-vimentin antibody (1:1000, ab137321, Abcam, Cambridge, UK) at 37 °C for 1 h. The sections were subsequently stained with Alexa Fluor 488 goat anti-rabbit IgG antibody (1:1000, A-11008, Invitrogen) at 37 °C for 30 min, stained with 4′,6-diamidino-2-phenylindole (DAPI; Sigma-Aldrich) for 5 min and observed under a fluorescence microscope (Leica Microsystems) for the determination of UC-MSCs. The UC-MSC density was evaluated by the number of cells positive for CM-Dil and vimentin counted from six randomly selected fields per section under a magnification of × 600.

### Histological analysis

As shown in Additional file 1: Figure S1, at days 30 and 60 post-transplantation, a subset of rats (*n* = 8 uterine horns) from each group was euthanized and the operative region of each uterine horn was dissected, fixed in 10% buffered formalin solution for 24 h, dehydrated in graded alcohols and embedded in paraffin perpendicularly. Sections (2 μm) of uterine horns were sliced transversally.

Haematoxylin-eosin staining was applied to observe the tissue structure and Masson’s trichrome staining was used to reveal collagen deposition. For immunohistochemistry, sections were stained with the anti-matrix metalloproteinase-2 antibody (MMP-2; 1:300, ab37150, Abcam), the anti-matrix metalloproteinase-9 antibody (MMP-9; 1:1000, ab76003, Abcam), the anti-α-smooth muscle actin antibody (α-SMA; 1:400, ab5694, Abcam) and the anti-von Willebrand factor antibody (vWF; 1:10,000, ab6994, Abcam). MMP-9 expression was evaluated by the number of cells positive for MMP-9 counted from six randomly selected fields per section under a magnification of × 400, covering most of the scarred areas. Smooth muscle abundance was evaluated by the percentage of α-SMA-positive areas (α-SMA positive area in the selected region/total α-SMA positive area) by Image-Pro Plus software (Media Cybernetics, Inc., Rockville, MD, USA). The blood vessel density was evaluated by the number of capillary vessels counted from six randomly selected fields per section under a magnification of × 400.

### Immunofluorescence analysis

To determine the types of cells expressing MMP-9, part of the slides that were prepared in the tracking of transplanted UC-MSCs were stained with the anti-MMP-9 antibody (1:200, ab76003, Abcam) and observed under a fluorescence microscope (Leica Microsystems).

### Western blotting

Endometrial tissues in the mid-secretory phase were obtained from women with normal menstrual cycles by endometrial biopsy at the Drum Tower Hospital Affiliated to Nanjing Medical University. Informed consent was obtained. Protocols for sampling endometrial tissues were approved by the Ethics Review Board of the Drum Tower Hospital Affiliated to Nanjing Medical University. Human endometrial stromal cells (hESCs) were isolated from endometrial tissues as previously described [42]. The expression of MMP-9 in hESCs, UC-MSCs in the monolayer culture (2D culture), UC-MSCs co-cultured with degradable collagen fibres (3D culture) was analysed by Western blotting. Serum-free medium served as the negative control and Ishikawa cells served as the positive control. Approximately 3 × 10^5^ cells were seeded onto 60-mm cell culture dishes. In 3D culture, UC-MSCs were co-cultured with degradable collagen fibres (0.56 mg/ml). After culturing for 72 h, the cells were switched to serum-free medium. After 48 h, culture medium was collected and centrifuged at 1000 rpm for 5 min. The cells were rinsed twice with pre-cooled PBS, after which 400 μL cell lysis buffer (50.0 mmol/L Tris pH = 7.6, 150.0 mmol/L NaCl, 0.1% SDS, 1.0% NP-40, protease inhibitor cocktail) was added and the cells were scraped off. The cells were lysed at 4 °C for 30 min under rotation and centrifuged at 15,000 rpm for 10 min and the supernatant was collected. Protein concentrations were determined by the BCA Protein Assay Reagent (Thermo Fisher Scientific, Rockford, IL, USA). Equal amounts of protein from cell lysates and culture medium were subjected to Western blot analysis and probed with the anti-MMP-9 antibody (1:10,000, ab76003, Abcam). To ensure equal protein loading, blots were probed with β-actin (1:10,000, ab8226, Abcam) and stained with Ponceau.

### Fertility test

The function of the scarred uterine horns was assessed by testing whether they were capable of receiving fertilized ova and supporting embryos to the late stage of pregnancy. At day 60 post-transplantation, another subset of rats (*n* = 16 uterine horns) from each group was mated with proven fertile male Sprague-Dawley rats. The rats were euthanized 18 days after the presence of vaginal plugs, and each uterine horn was examined for numbers, sizes and weights of fetuses, as well as sites of implantation.

### Statistical analysis

Histological measurements were performed by two independent observers and an average was taken for the subsequent analysis. Histological data were presented as mean ± standard deviation (SD), and multiple group comparisons were determined by one-way ANOVA. The numbers of fetuses were presented as median and minimum and maximum, and multiple group comparisons were determined by Kruskal-Wallis test and Nemenyi test. The pregnancy rates were presented as count and percentage, and multiple group comparisons were analysed by Fisher exact test followed by Bonferroni correction. Statistics were calculated using IBM SPSS Statistics Package for Social Science (Version 22.0, IBM Corp., Armonk, NY, USA). *P* < 0.05 was considered statistically significant.

## Results

### Confirmation of uterine scar formation

**Fig. 2:**
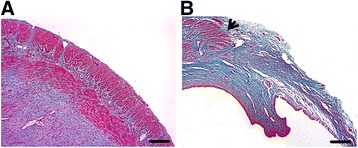
Confirmation of uterine scar formation. Thirty days after full-thickness excision of uterine walls, Masson’s trichrome staining of normal uterine tissue **a** and wounded uterine tissue **b**. *Arrowheads* indicate repair sites. Scale bars, 150 μm

Thirty days after full-thickness excision of uterine walls, Masson’s trichrome staining indicated that unlike normal uterine tissue (Fig. 2a), the wounded uterine tissue exhibited a thin uterine wall, in which normal endometrium and myometrium was mostly replaced with collagen deposits (Fig. 2b), validating the formation of uterine scars.

### UC-MSCs express specific surface antigens and possess multi-lineage differentiation potential

**Fig. 3:**
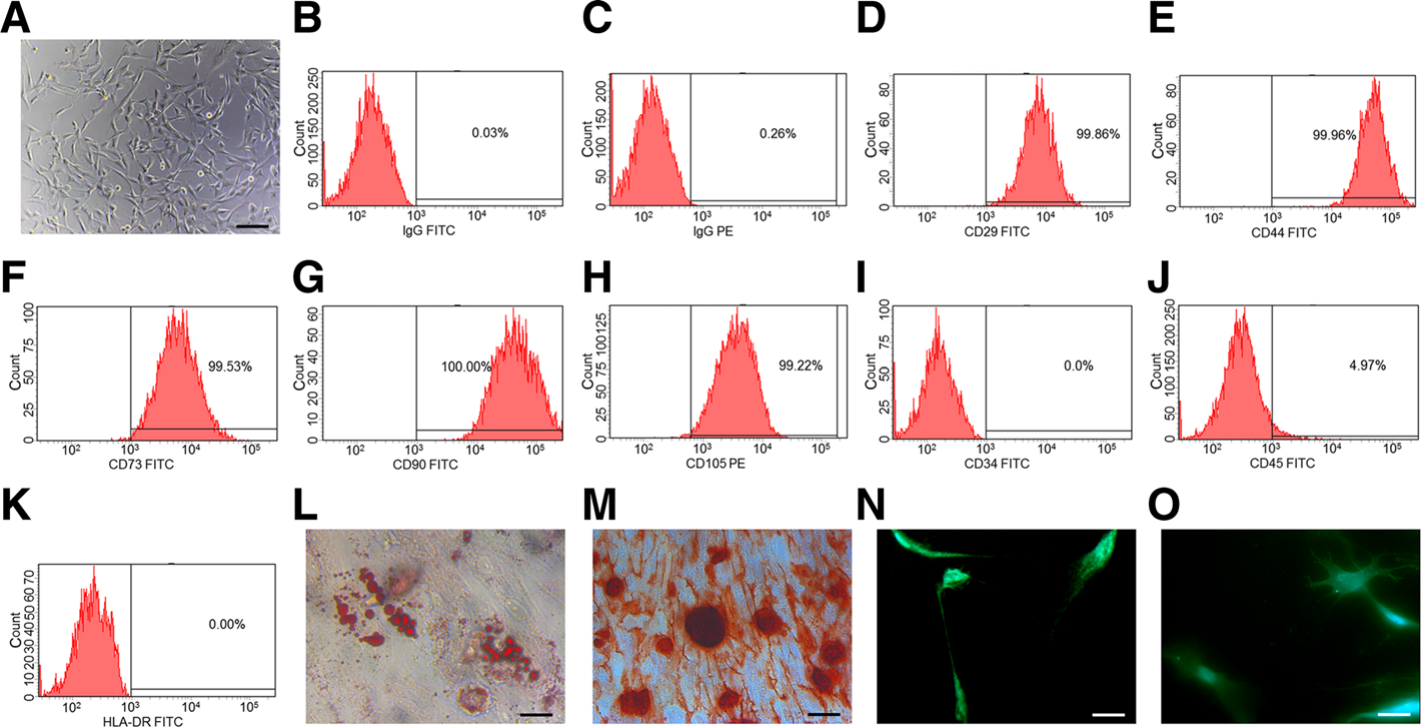
UC-MSCs express specific surface antigens and possess multi-lineage differentiation potential. **a** Morphology of human UC-MSCs. Scale bars, 30 μm. **b**-**k** Flow cytometry analysis of immune-markers in human UC-MSCs. **l**-**o** Differentiation assays of human UC-MSCs. Scale bars, 10 μm. Adipogenesis was confirmed by Oil red O staining to show intracellular lipid accumulation (**l**). Osteogenesis was confirmed by Alizarin red staining to show calcium deposition (**m**). Neural-like differentiation was confirmed by immunofluorescence staining with anti-NSE antibody (**n**) and anti-NF-M antibody (**o**). *FITC* fluorescein isothiocyanate, *PE* phycoerythrin

According to the characteristics of MSCs defined by the International Society for Cellular Therapy [43], UC-MSCs were checked for adherence to plastic, specific surface antigen expression and multipotent differentiation potential. After culturing human umbilical cord tissues for 14 days, spindle-shaped adherent cells were apparent (Fig. 3a). These cells were positive for CD29, CD44, CD73, CD90 and CD105, and were negative for CD34, CD45 and HLA-DR (Fig. 3b–k). Moreover, these cells displayed the capacity to differentiate into adipocytes, osteoblasts and neural-like cells after induction in vitro (Fig. 3 l–o), indicating their multi-lineage differentiation potential.

### Scaffolds promote the long-term retention of UC-MSCs in uterine scars

**Fig. 4:**
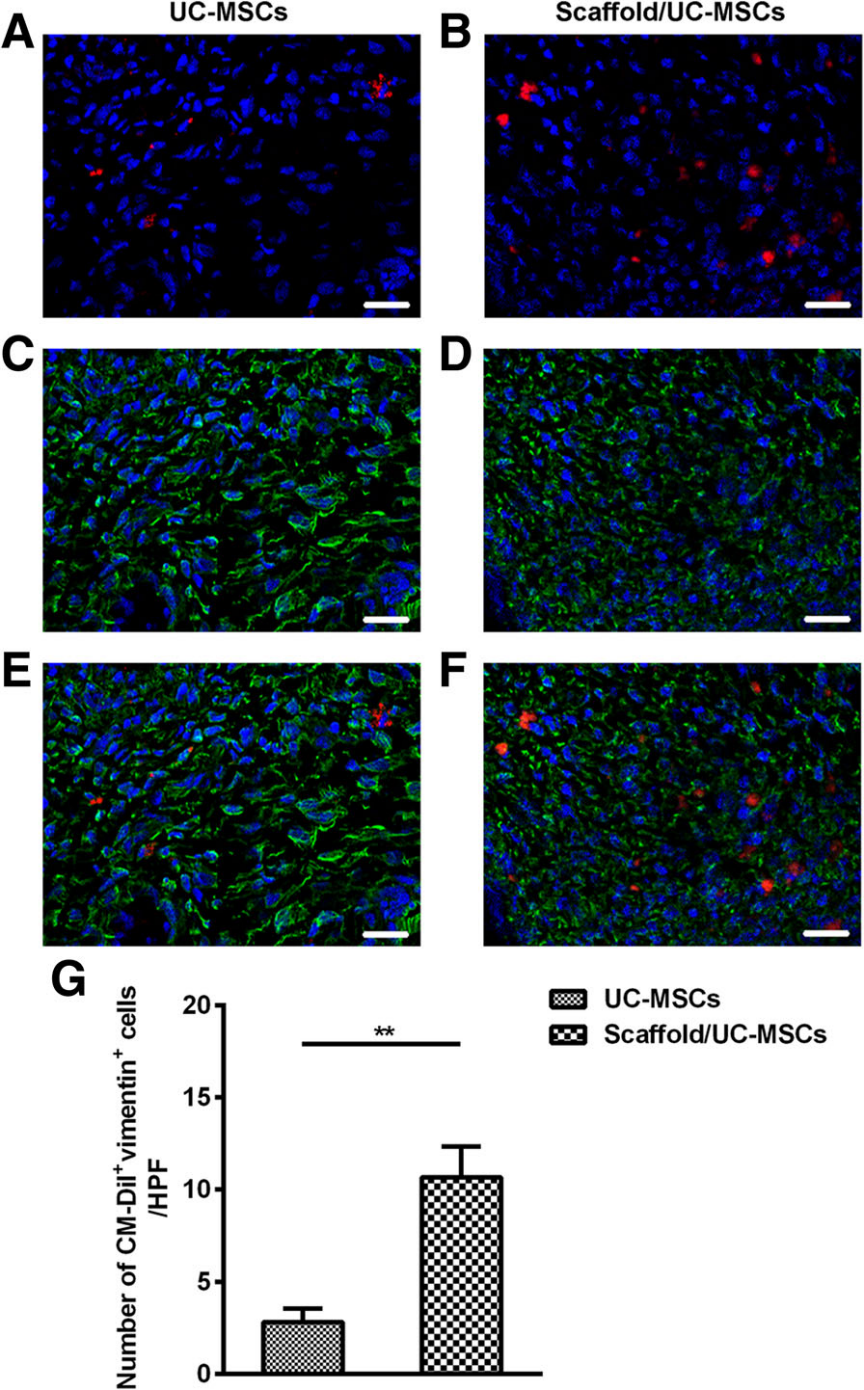
Scaffolds promote the long-term retention of UC-MSCs in uterine scars. **a**, **b** Uterine scars were transplanted with CM-Dil-labelled UC-MSCs or scaffold/UC-MSCs. At day 30 post-transplantation, uterine tissues containing the scarred areas were collected, embedded and sectioned. Cell nuclei were stained with DAPI (*blue*). UC-MSCs were tracked by the *red* fluorescence of CM-Dil under a fluorescence microscope. **c**-**f** The sections were stained with anti-vimentin antibody and observed under a fluorescence microscope (*green*). Scale bars, 20 μm. **g** Statistical analysis of the number of cells positive for CM-Dil and vimentin counted from six randomly selected fields per section under a magnification of × 600. ^*^ *P* < 0.05 and ^**^ *P* < 0.01. *UC-MSCs* umbilical cord-derived mesenchymal stem cells

At day 30 post-transplantation, labelled UC-MSCs were found to mainly distribute in the stroma of the scarred uterine walls. Significantly more labelled UC-MSCs were observed in the stroma of the scaffold/UC-MSCs group than in the UC-MSCs group (Fig. 4a, b). Moreover, the CM-Dil-labelled UC-MSCs were positive for vimentin, a signature marker for MSCs (Fig. 4c–f). The number of cells positive for CM-Dil and vimentin in the scaffold/UC-MSCs group (10.67 ± 1.67) was significantly higher than that in the UC-MSCs group (2.83 ± 0.75, *P* < 0.01) (Fig. 4 g). These results indicate that scaffolds are helpful in limiting the diffusion of transplanted UC-MSCs in the scarred areas.

### Scaffold/UC-MSCs transplantation facilitates collagen degradation in uterine scars via upregulation of MMP-9

**Fig. 5:**
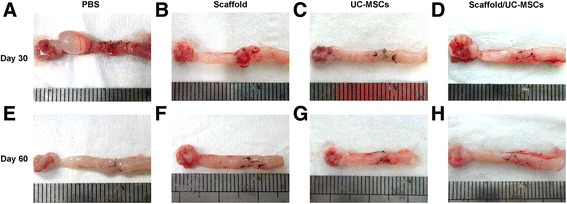
Morphology of uterine scars following different treatments. Gross view of uterine scars at days 30 and 60 post-transplantation in the PBS group (**a**, **e**), the scaffold group (**b**, **f**), the UC-MSCs group (**c**, **g**) and the scaffold/UC-MSCs group (**d**, **h**). *PBS* phosphate-buffered saline, *UC-MSCs* umbilical cord-derived mesenchymal stem cells

Gross examination at day 30 post-transplantation revealed pale appearance without obvious angiogenesis, contractures and excrescences in uterine scars treated with PBS, scaffolds or UC-MSCs. In addition, two PBS-treated uterine horns developed distal hydrometra caused by intratubal obstruction. However, the scaffold/UC-MSCs group exhibited obvious neovascularization and no apparent excrescences or contractures in the scarred areas (Fig. 5a-d). At day 60 post-transplantation, more apparent contractures and excrescences were observed in uterine scars treated with PBS, scaffolds or UC-MSCs; while the scaffold/UC-MSCs-treated uterine scars were similar to normal tissues on inspection and in texture (Fig. 5e-h).

**Fig. 6:**
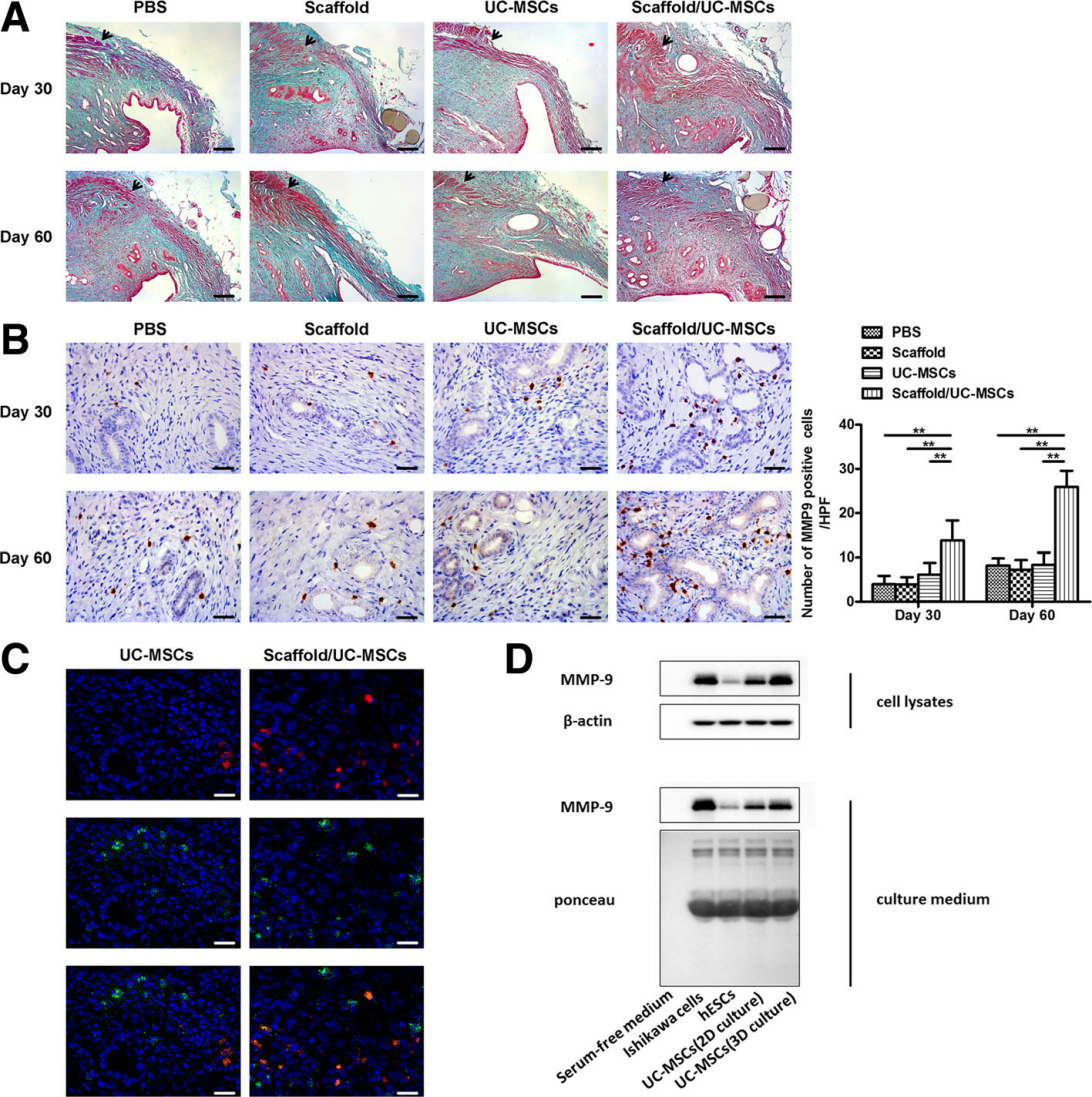
Scaffold/UC-MSCs transplantation facilitates collagen degradation in uterine scars via upregulation of MMP-9. **a** Masson’s trichrome staining of uterine scars at days 30 and 60 post-transplantation in the PBS group, the scaffold group, the UC-MSCs group and the scaffold/UC-MSCs group. *Arrowheads* indicate repair sites. Scale bars, 150 μm. **b** Immunohistochemical staining of matrix metalloproteinase-9 (MMP-9) in uterine scars at days 30 and 60 post-transplantation in the PBS group, the scaffold group, the UC-MSCs group and the scaffold/UC-MSCs group. Scale bars, 30 μm. Statistical analysis of the number of cells positive for MMP-9 counted from six randomly selected fields per section under a magnification of × 400. Data were presented as mean ± SEM. ^*^ *P* < 0.05 and ^**^ *P* < 0.01. **c** Uterine scars were transplanted with CM-Dil-labelled UC-MSCs or scaffold/UC-MSCs. At day 30 post-transplantation, uterine tissues containing the scarred areas were collected, embedded and sectioned. Cell nuclei were stained with DAPI (*blue*). UC-MSCs were tracked by the *red* fluorescence of CM-Dil. The sections were stained with anti-MMP-9 antibody and observed under a fluorescence microscope (*green*). Scale bars, 20 μm. **d** Western blot analysis of MMP-9 protein in cell lysates and culture medium of human endometrial stromal cells (hESCs), UC-MSCs in the monolayer culture (2D culture), UC-MSCs co-cultured with degradable collagen fibres (3D culture). Serum-free medium served as the negative control and Ishikawa cells served as the positive control. Blots were probed with β-actin and stained with Ponceau to ensure equal protein loading and transfer. *PBS* phosphate-buffered saline, *UC-MSCs* umbilical cord-derived mesenchymal stem cells

To assess the fibrosis in uterine scars, Masson’s trichrome staining was performed. At day 30 post-transplantation, uterine scars in the PBS group, the scaffold group and the UC-MSCs group showed abundant collagen deposition and a massive loss of native cells. However, the scaffold/UC-MSCs group had obvious collagen degradation and apparent regenerated endometrial glands and muscle bundles (Fig. 6a). At day 60 post-transplantation, uterine scars in the PBS group, the scaffold group and the UC-MSCs group did not show apparent collagen degradation compared with day 30 post-transplantation. Nevertheless, collagen fibres in the scaffold/UC-MSCs group further decreased; while the endometrium and myometrium regenerated (Fig. 6a).

In the uterus, collagen degradation mainly involves matrix metalloproteinase-2 (MMP-2) and matrix metalloproteinase-9 (MMP-9) [44, 45]. MMP-9 expression was detected in the endometrial stroma. At day 30 post-transplantation, the number of MMP-9-positive cells in the scaffold/UC-MSCs group was significantly higher than that in the other three groups (Fig. 6b). At day 60 post-transplantation, although increased in all groups compared with day 30 post-transplantation, the number of MMP-9-positive cells in the scaffold/UC-MSCs group (25.96 ± 3.63) remained higher than that the PBS group (8.19 ± 1.61, *P* < 0.01), the scaffold group (7.25 ± 2.17, *P* < 0.01) and the UC-MSCs group (8.31 ± 2.77, *P* < 0.01) (Fig. 6b). MMP-2 was immunostained in the endometrial stroma and glandular epithelium, and there was no significant difference in its expression among the four groups (Additional file 2: Figure S2).

Immunofluorescence staining further revealed the pattern of MMP-9 expression at day 30 post-transplantation. In both the UC-MSCs group and the scaffold/UC-MSCs group, only a small proportion of endometrial stroma cells expressed MMP-9; while almost all transplanted UC-MSCs labelled by CM-Dil expressed MMP-9. Furthermore, with the help of scaffolds, more UC-MSCs were located in the stroma of the scaffold/UC-MSCs group than the UC-MSCs group. As a result, the scaffold/UC-MSCs group had significantly more positive staining for MMP-9 compared with the UC-MSCs group, which is inconsistent with the immunohistochemistry results (Fig. 6c). Western blot analysis of cell lysates and culture medium was performed to measure the amount of protein expressed and released into the extracellular environment by human endometrial stromal cells (hESCs), hUC-MSCs in the monolayer culture (2D culture) and hUC-MSCs co-cultured with degradable collagen fibres (3D culture). Increased expression of cellular and extracellular MMP-9 was detected in hUC-MSCs relative to hESCs. Moreover, compared with 2D culture, 3D culture significantly increased the production and secretion of MMP-9 by hUC-MSCs (Fig. 6d).

### Scaffold/UC-MSCs transplantation promotes regeneration of the endometrium, myometrium and blood vessels in uterine scars

**Fig. 7:**
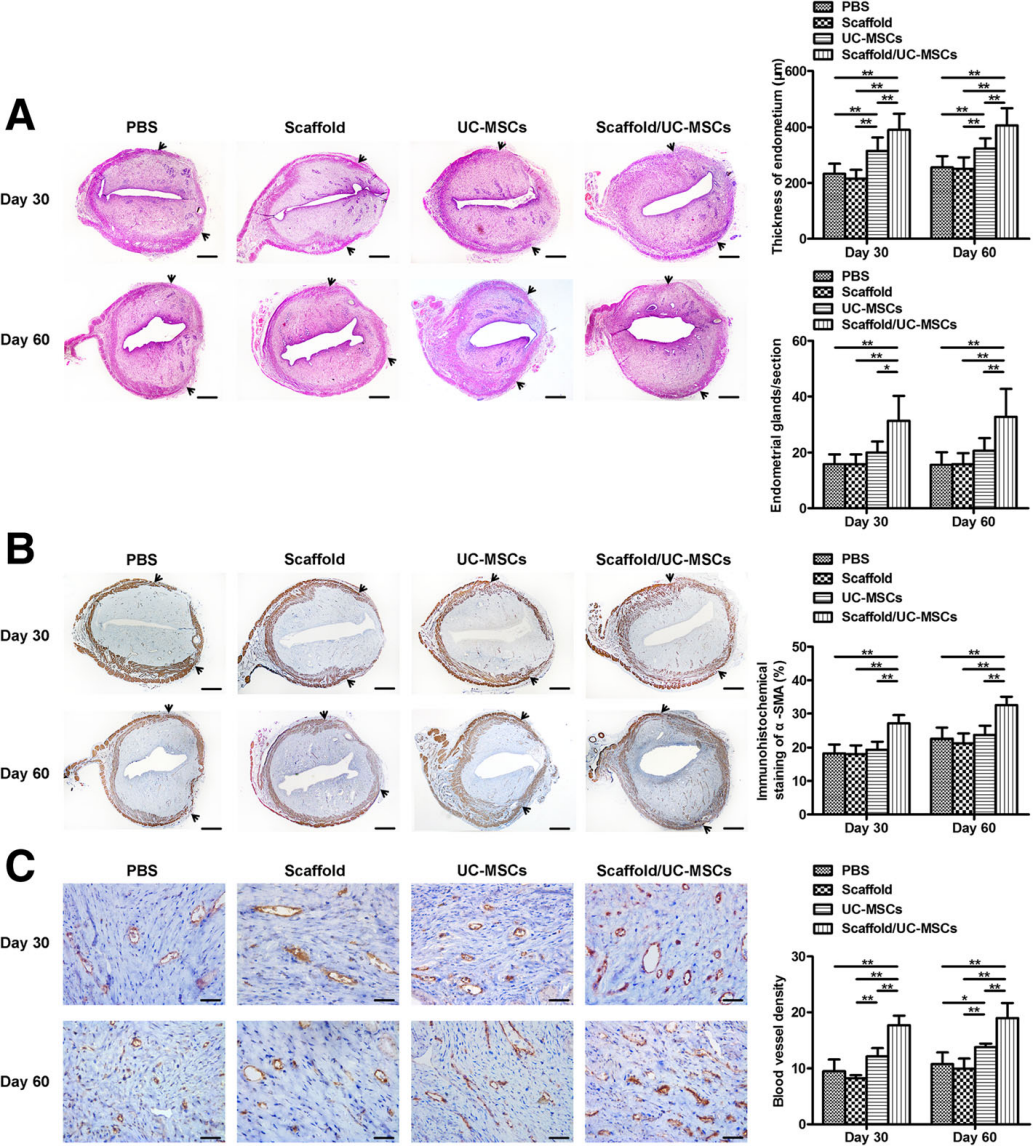
Scaffold/UC-MSCs transplantation promotes regeneration of the endometrium, myometrium and blood vessels in uterine scars. **a** Haematoxylin and eosin (H&E) staining of uterine scars at days 30 and 60 post-transplantation in the PBS group, the scaffold group, the UC-MSCs group and the scaffold/UC-MSCs group. *Arrowheads* indicate repair sites. Scale bars, 300 μm. Statistical analysis of the endometrial thickness measured by Image-Pro Plus software. Statistical analysis of the number of endometrial glands per uterine cross section. **b** Immunohistochemical staining of α-smooth muscle actin (α-SMA) for smooth muscle abundance in uterine scars at days 30 and 60 post-transplantation in the PBS group, the scaffold group, the UC-MSCs group and the scaffold/UC-MSCs group. *Arrowheads* indicate repair sites. Scale bars, 300 μm. Statistical analysis of the percent of α-SMA positive areas (α-SMA-positive area in the selected region/total α-SMA-positive area) by Image-Pro Plus software. **c** Immunohistochemical staining of von Willebrand factor (vWF) for the blood vessel density in uterine scars at days 30 and 60 post-transplantation in the PBS group, the scaffold group, the UC-MSCs group and the scaffold/UC-MSCs group. Scale bars, 30 μm. Statistical analysis of the number of capillary vessels counted from six randomly selected fields per section under a magnification of × 400. Data were presented as mean ± SEM. ^*^ *P* < 0.05 and ^**^ *P* < 0.01. *PBS* phosphate-buffered saline, *UC-MSCs* umbilical cord-derived mesenchymal stem cells

To evaluate the effect of the scaffold/UC-MSCs construct on regeneration of the endometrium during collagen degradation, endometrial thickness and the number of endometrial glands were assessed by H&E staining. As shown in Fig. 7a, at day 30 post-transplantation, the thickness of the endometrium in the scaffold/UC-MSCs group was significantly higher than that in the other three groups. At day 60 post-transplantation, the scaffold/UC-MSCs group (406.87 ± 61.39 μm) continued to exhibit thicker endometria than the PBS group (256.25 ± 40.31 μm, *P* < 0.01), the scaffold group (250.06 ± 41.86 μm, *P* < 0.01) and the UC-MSCs group (323.92 ± 35.53 μm, *P* < 0.01). The endometrial thickness in the UC-MSCs group was significantly higher than that in the PBS group (*P* < 0.01) and the scaffold group (*P* < 0.01) (Fig. 7a). Furthermore, there were more endometrial glands per uterine cross section in the scaffold/UC-MSCs group (32.88 ± 9.89) compared to the PBS group (15.63 ± 4.50, *P* < 0.01), the scaffold group (15.88 ± 3.98, *P* < 0.01) and the UC-MSCs group (20.75 ± 4.50, *P* < 0.01) (Fig. 7a).

Apart from the endometrium, the myometrium is another important component of the uterine wall. As shown in Fig. 7b, in the scaffold/UC-MSCs group, muscle bundles formed a thin continuous layer beneath the endometrium with circular fibres at day 30 post-transplantation. However, in the other groups, the muscle layers were thin and discontinuous. The percentage of α-SMA-positive areas in the scaffold/UC-MSCs group was significantly higher than that in the other three groups (Fig. 7b). At day 60 post-transplantation, an increase in muscle bundles was observed in each group compared with the early stage. Muscle layers in the PBS group, the scaffold group and the UC-MSCs group remained thin and disordered. However, muscle bundles in the scaffold/UC-MSCs group were well organized with a new separate muscle layer, which were similar to normal tissues (Fig. 7b). The percentage of α-SMA-positive areas in the scaffold/UC-MSCs group (32.55% ± 2.49%) far exceeded that in the PBS group (22.61% ± 3.23%, *P* < 0.01), the scaffold group (21.32% ± 2.80%, *P* < 0.01) and the UC-MSCs group (23.70% ± 2.65%, *P* < 0.01) (Fig. 7b).

Re-establishment of blood supply is fundamental for regeneration of uterine scars. Quantitative analysis at day 30 post-transplantation confirmed a higher blood vessel density in the scaffold/UC-MSCs group than the other three groups (Fig. 7c). At day 60 post-transplantation, the blood vessel density increased in all groups compared with day 30 post-transplantation, but continued to be higher in the scaffold/UC-MSCs group (18.98 ± 2.66) than in the PBS group (10.71 ± 2.11, *P* < 0.01), the scaffold group (9.96 ± 1.76, *P* < 0.01) and the UC-MSCs group (13.71 ± 0.70, *P* < 0.01). The blood vessel density in the UC-MSCs group was significantly higher than that in the PBS group (*P* < 0.05) and the scaffold group (*P* < 0.01) (Fig. 7c).

### Scaffold/UC-MSCs transplantation restores receptive fertility of uterine scars

**Fig. 8:**
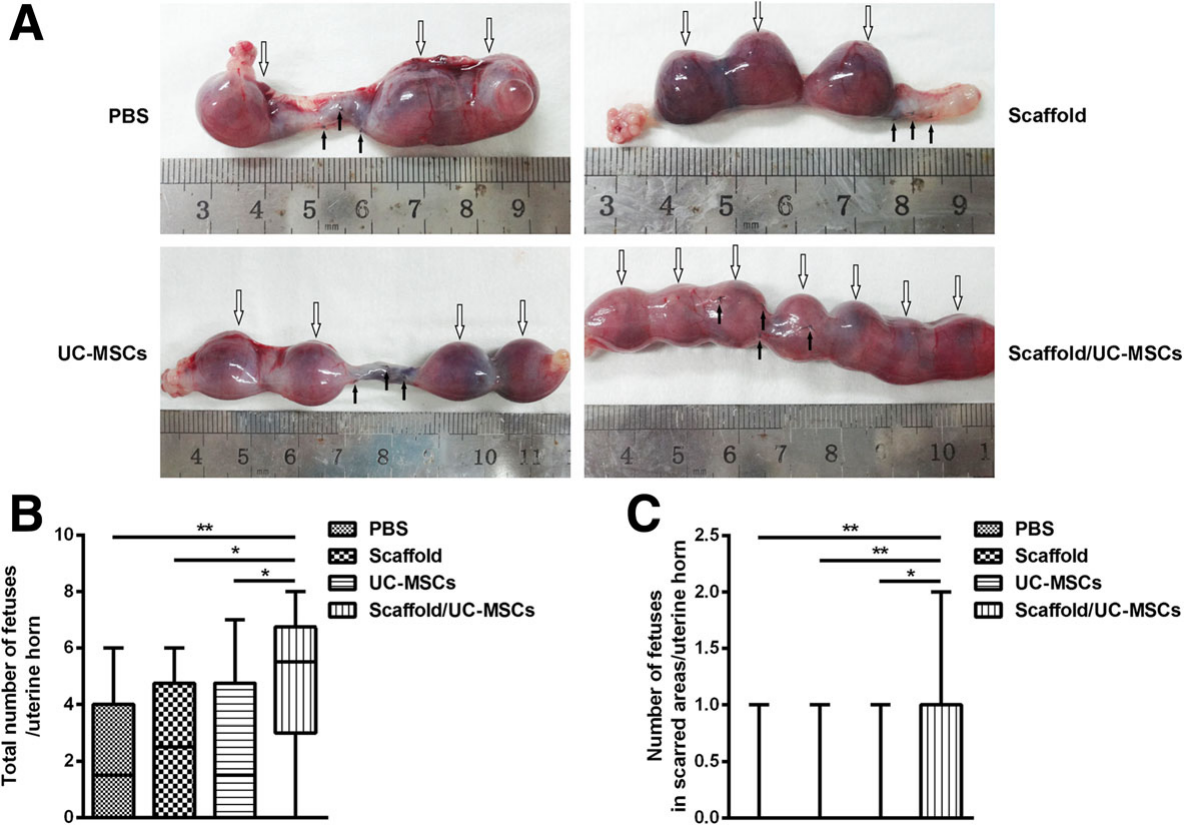
Scaffold/UC-MSCs transplantation restores receptive fertility of uterine scars. **a** Pregnancies at day 60 post-transplantation in the PBS group, the scaffold group, the UC-MSCs group and the scaffold/UC-MSCs group. *Hollow arrows* indicate implanted fetuses and *black arrows* indicate the four pre-marked points surrounding each uterine scar. **b** Statistical analysis of the total number of fetuses per uterine horn. **c** Statistical analysis of the number of fetuses implanted within the scarred areas per uterine horn. Data were presented as median and minimum and maximum. ^*^ *P* < 0.05 and ^**^ *P* < 0.01. *PBS* phosphate-buffered saline, *UC-MSCs* umbilical cord-derived mesenchymal stem cells

At day 60 post-transplantation, implanted fetuses were found in some of the uterine horns, which were maintained up to the late viable stage of pregnancy. The total number of fetuses per uterine horn in the scaffold/UC-MSCs group (5.50, range 0–8) was significantly higher than that in the PBS group (1.50, range 0–6, *P* < 0.01), the scaffold group (2.50, range 0–6, *P* < 0.05) and the UC-MSCs group (1.50, range 0–7, *P* < 0.05) (Fig. 8a, b). The pregnancy rate in the scaffold/UC-MSCs group was higher than that in the other three groups, although there were no statistical differences in the pregnancy rate among the four groups (Table 1). Further analysis of implantation sites demonstrated that the number of fetuses implanted within the scarred areas per uterine horn in the scaffold/UC-MSCs group (1, range 0–2) was significantly higher than that in the PBS group (0, range 0–1, *P* < 0.01), the scaffold group (0, range 0–1, *P* < 0.01) and the UC-MSCs group (0, range 0–1, *P* < 0.05) (Fig. 8c). The pregnancy rate in the scaffold/UC-MSCs group was significantly higher than that in the other three groups (Table 1). Furthermore, fetuses implanted within the scarred areas had normal sizes and weights compared with fetuses implanted in the non-scarred areas (data not shown). The results above imply complete restoration of receptive fertility of scaffold/UC-MSCs-treated uterine scars.

**Table 1:** Comparison of reproductive outcomes among different treatments at day 60 post-transplantation^a^

Variable	PBS group	Scaffold group	UC-MSCs group	Scaffold/UC-MSCs group	*P* value
Total number of uterine horns	16	16	16	16	
Pregnant uterine horns (%)	10 (62.50%)	10 (62.50%)	11 (68.75%)	15 (93.75%)	>0.05
Uterine horns with fetus implantation in scarred areas (%)	2 (12.50%)^b^	1 (6.25%)^c^	3 (18.75%)^d^	10 (62.50%)	<0.05

*PBS* phosphate-buffered saline, *UC-MSCs* umbilical cord-derived mesenchymal stem cells

^a^Bonferroni correction: critical level of significance, *P* < 0.017

^b^ *P* < 0.017, PBS group versus scaffold/UC-MSCs group

^c^ *P* < 0.017, scaffold group versus scaffold/UC-MSCs group

^d^ *P* < 0.017, UC-MSCs group versus scaffold/UC-MSCs group

## Discussion

In the present study, we demonstrated for the first time that the scaffold/UC-MSCs system facilitated collagen degradation, full-thickness regeneration and fertility restoration in uterine scars; and the underlying mechanism of which might rely on the long-term effect of UC-MSCs in vivo.

In uterine scars, collagen deposition is a major clinical problem, which impedes the proliferation, differentiation and migration of uterine native cells. Efficient collagen degradation in uterine scars treated with scaffold/UC-MSCs compared to uterine scars treated with PBS, scaffolds or UC-MSCs was demonstrated in the present study. In the early stage of postmenstrual repair of the endometrium, which represents the only example of cyclic scar-free repair in adult human tissue, expression of MMPs, including MMP-2 and MMP-9 is elevated both in vivo and in vitro, suggesting the essential role of MMPs in preventing the formation of uterine scars [44]. In our study, the substantial increase in MMP-9 expression induced by the scaffold/UC-MSCs construct in uterine scar tissues, which was validated by immunohistochemistry and immunofluorescence staining, might represent a possible explanation for the reduced collagen deposition. Moreover, we found that the co-culture with degradable collagen fibres promoted the production and secretion of MMP-9 by UC-MSCs in vitro compared with the monolayer culture. This is inconsistent with our previous observation of an increased expression of paracrine factors in adipose-derived mesenchymal stem cells co-cultured with degradable collagen fibres compared with the monolayer culture [41]. When mixed with degradable collagen fibres, UC-MSCs interacted with collagen fibres to form a 3D microenvironment, which provided a suitable niche for UC-MSCs to anchor, migrate and function.

Besides increased collagen degradation, facilitated regeneration of endometrial endometrium, glandular epithelium, myometrium and blood vessels was also observed in the scaffold/UC-MSCs-treated uterine scars. In the process of endometrial cyclic regeneration, large quantities of cytokines, such as FGF2 and VEGF, have been demonstrated to be essential [46, 47]. It has been proven by protein chips that UC-MSCs express paracrine factors including FGF2 and VEGF (data not shown), which is consistent with the reported secretion of FGF2 and VEGF by UC-MSCs [48, 49]. Furthermore, the 3D culture increased the expression of FGF2 and VEGF by UC-MSCs compared with the 2D culture (data not shown). In the present study, as the distribution of transplanted UC-MSCs was proven to be in the endometrial stroma, it was possible that the paracrine mechanism was involved in regeneration of the endometrial glandular epithelium, myometrium and blood vessels.

The most important function of the uterus is to establish and maintain pregnancy. Contributing from structural reconstruction, the scaffold/UC-MSCs-treated uterine scars showed nearly complete restoration of receptive fertility.

## Conclusions

In this study, we demonstrated in a rat model that the scaffold/UC-MSCs system could facilitate collagen degradation in uterine scars via upregulation of MMP-9, which mainly derived from the transplanted UC-MSCs, and promoted regeneration of the endometrium, myometrium and blood vessels in uterine scars. Contributing from structural reconstruction, the scaffold/UC-MSCs-treated uterine scars showed improved pregnancy outcomes. Overall, scaffold/UC-MSCs transplantation may be a novel therapeutic approach for the treatment of uterine scars.

## Additional files

Additional file 1: Figure S1. The study design flowchart. In order to investigate the effect of different treatments on the structure and function of uterine scars, 128 scarred uterine horns from 64 rats were randomly assigned to four groups, including a PBS group (*n* = 32 uterine horns), scaffold group (*n* = 32 uterine horns), UC-MSCs group (*n* = 32 uterine horns) and scaffold/UC-MSCs group (*n* = 32 uterine horns). In addition, in order to track the transplanted UC-MSCs in the scarred areas, eight scarred uterine horns from four rats were randomly assigned to two groups, including a UC-MSCs group (*n* = 4 uterine horns) and scaffold/UC-MSCs group (*n* = 4 uterine horns). (TIF 495 kb)

Additional file 2: Figure S2. No significant difference in MMP-2 expression is observed among the four groups. Immunohistochemical staining of MMP-2 in uterine scars at days 30 and 60 post-transplantation in the PBS group (**A**, **E**), the scaffold group (**B**, **F**), the UC-MSCs group (**C**, **G**) and the scaffold/UC-MSCs group (**D**, **H**). Scale bars, 30 μm. (TIF 2961 kb)

## Abbreviations

ATRA: All-trans-retinoic acid
BM-MSCs: Bone marrow-derived mesenchymal stem cells
BSA: Bovine serum albumin
CBD: Collagen-binding domain
DAPI: 4′,6-diamidino-2-phenylindole
FBS: Fetal bovine serum
FGF2: Fibroblast growth factor 2
FITC: Fluorescein isothiocyanate
hESCs: Human endometrial stromal cells
IGF1: Insulin-like growth factor 1
MMP-2: Matrix metalloproteinase-2
MMP-9: Matrix metalloproteinase-9
OCT: Optimal cutting temperature compound
PBS: Phosphate-buffered saline
PE: Phycoerythrin
SD: Sprague-Dawley
SD: Standard deviation
UC-MSCs: Umbilical cord-derived mesenchymal stem cells
VEGF: Vascular endothelial growth factor
vWF: von Willebrand factor
α-SMA: α-smooth muscle actin
