# Supplementary figures and images for: Umbilical cord-derived mesenchymal stem cells on scaffolds facilitate collagen degradation via upregulation of MMP-9 in rat uterine scars
Source: Stem Cell Res Ther. 2017 Apr 18;8:84. doi: 10.1186/s13287-017-0535-0 (PMC5395893; doi:10.1186/s13287-017-0535-0)

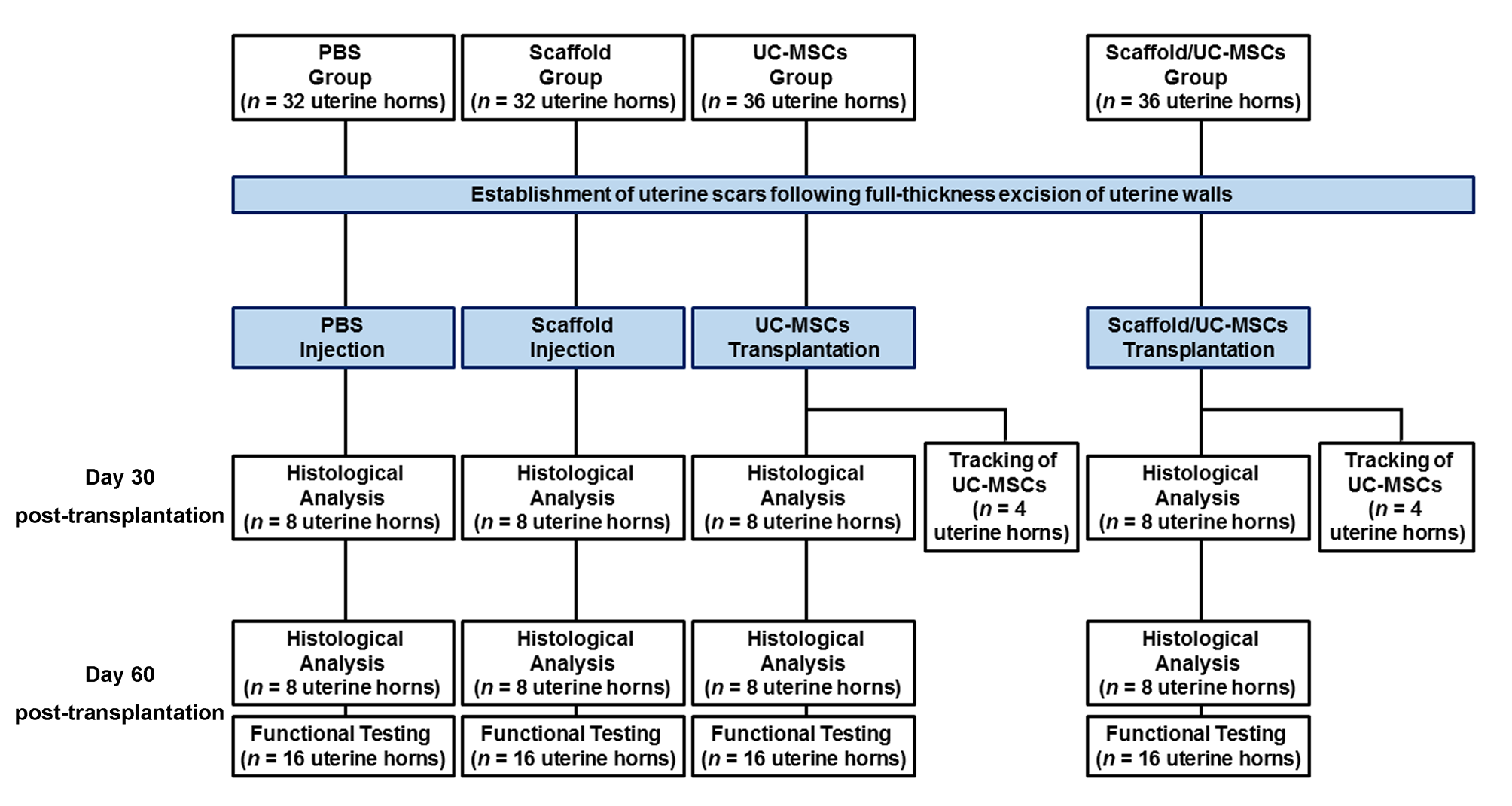

Supplement: Supplementary file 1 — The study design flowchart. In order to investigate the effect of different treatments on the structure and function of uterine scars, 128 scarred uterine horns from 64 rats were randomly assigned to four groups, including a PBS group (n = 32 uterine horns), scaffold group (n = 32 uterine horns), UC-MSCs group (n = 32 uterine horns) and scaffold/UC-MSCs group (n = 32 uterine horns). In addition, in order to track the transplanted UC-MSCs in the scarred areas, eight scarred uterine horns from four rats were randomly assigned to two groups, including a UC-MSCs group (n = 4 uterine horns) and scaffold/UC-MSCs group (n = 4 uterine horns). (TIF 495 kb) [file 13287_2017_535_MOESM1_ESM.tif]

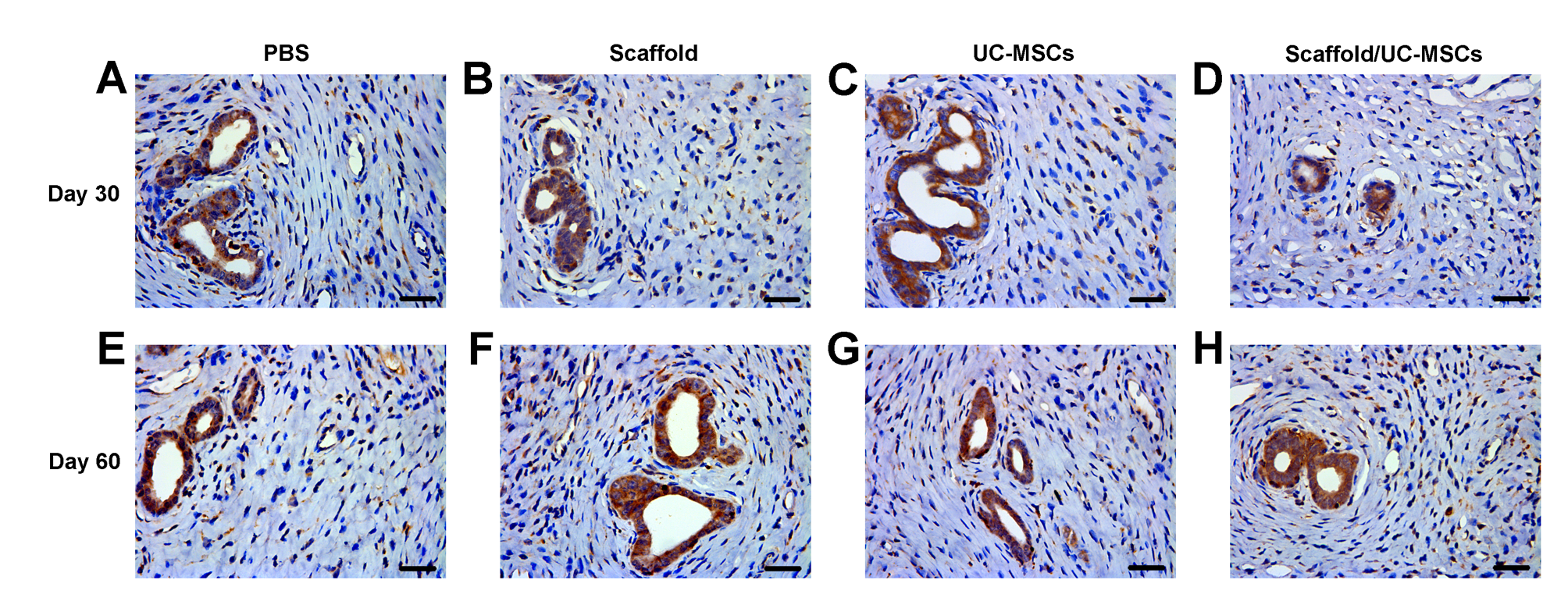

Supplement: Supplementary file 2 — No significant difference in MMP-2 expression is observed among the four groups. Immunohistochemical staining of MMP-2 in uterine scars at days 30 and 60 post-transplantation in the PBS group (A, E), the scaffold group (B, F), the UC-MSCs group (C, G) and the scaffold/UC-MSCs group (D, H). Scale bars, 30 μm. (TIF 2961 kb) [file 13287_2017_535_MOESM2_ESM.tif]
